# Safety monitoring of treatment in bipolar disorder in a tertiary care setting in Sri Lanka and recommendations for improved monitoring in resource limited settings

**DOI:** 10.1186/s12888-019-2183-7

**Published:** 2019-06-24

**Authors:** Uthpali Mannapperuma, Priyadarshani Galappatthy, Raveendra Laal Jayakody, Jayan Mendis, Varuni Asanka de Silva, Raveen Hanwella

**Affiliations:** 1 Department of Pharmacology and Pharmacy, Faculty of Medicine, University of Colombo, P.O.Box 271, Kynsey Road, Colombo 8, Sri Lanka; 2 National Institute of Mental Health, Angoda, Sri Lanka; 3 Department of Psychiatry, Faculty of Medicine, University of Colombo, Colombo 8, Sri Lanka

**Keywords:** Bipolar disorder, ISBD, Safety monitoring, Clinical audit

## Abstract

**Background:**

Safety monitoring of medicines is essential during therapy for bipolar disorder (BD). We determined the extent of safety monitoring performed according to the International Society for Bipolar Disorders (ISBD) guidelines in patients with BD attending the main tertiary care psychiatry clinics in Sri Lanka to give realistic recommendations for safety monitoring in resource limited settings.

**Methods:**

Patients diagnosed with BD on mood stabilizer medications for more than 1 year were recruited. Data were collected retrospectively from clinic and patient held records and compared with the standards of care recommended by ISBD guidelines for safety monitoring of medicines.

**Results:**

Out of 256 patients diagnosed with BD, 164 (64.1%) were on lithium. Only 75 (45.7%) had serum lithium measurements done in the past 6 months and 96 (58.5%) had concentrations recorded at least once in the past year. Blood urea or creatinine was measured in the last 6 months only in 30 (18.3%). Serum electrolytes and thyroid-stimulating hormone (TSH) concentrations were measured in the last year only in 34 (20.7%) and 30 (18.3%) respectively. Calcium concentrations were not recorded in any patient. None of the patients on sodium valproate (*n* = 119) or carbamazepine (*n* = 6) had blood levels recorded to establish therapeutic concentrations. Atypical antipsychotics were prescribed for 151 (59%), but only 13 (8.6%) had lipid profiles and only 31 (20.5%) had blood glucose concentration measured annually. Comorbidities experienced by patients influenced monitoring more than the medicines used. Patients with diabetes, hypothyroidism and hypercholesterolemia were more likely to get monitored for fasting blood glucose and (*p* < 0.001), TSH (p < 0.001) and lipid profiles (p < 0.001). Lithium therapy was associated with TSH monitoring (*p* < 0.05). Therapy with atypical antipsychotics was not associated with fasting blood glucose or lipid profile monitoring (*p* > 0.05). A limitation of the study is that although some tests were performed, the results may not have been recorded.

**Conclusions:**

Safety monitoring in BD was suboptimal compared to the ISBD guidelines. ISBD standards are difficult to achieve in resource limited settings due to a multitude of reasons. Realistic monitoring benchmarks and recommendations are proposed for methods to improve monitoring in resource limited settings based on our experience.

## Background

Long term maintenance treatment for bipolar disorder (BD) consists of using lithium, other mood stabilizers, antipsychotics and antidepressants [1]. Patients often require lifelong treatment to mitigate negative outcomes of the disease and to prevent relapses, hospitalization, functional impairment and suicide [2].

Safety monitoring is an essential component of therapy in BD and recommendations are given by several organizations such as the International Society for Bipolar Disorders (ISBD) [3], Canadian Network for Mood and Anxiety Treatments (CANMAT) and National Institute for Clinical Excellence (NICE), in the United Kingdom [1, 4, 5]. These recommendations come from high-income countries and even these countries sometimes have difficulty in meeting these recommendations given [6]. Therefore, more realistic targets without compromising patient care is needed, considering costs of monitoring and availability of human and other resources particularly for resource limited settings [7–10].

Those on lithium therapy require regular therapeutic drug monitoring as lithium has a narrow therapeutic index. Such monitoring minimizes lithium toxicity and can be used to assess adherence to medication [11]. Long-term treatment with lithium is associated with occurrence of renal side effects such as chronic kidney disease and renal failure. The renal side effects of lithium were evident both from clinical studies having small numbers of subjects and from large scale epidemiological studies [12–17]. Apart from the renal side effects, metabolic and cardiovascular effects are also described particularly with newer antipsychotics that are used in patients with BD [7, 10, 18]. These effects are associated with the prescribed medications as well as other factors [1, 18–21].

Clinical audits and descriptive studies are carried out to assess whether the quality of care provided to patients is in keeping with the recommended standards and guidelines [9]. The ISBD commissioned a working group to develop guidelines for safety monitoring in BD. The main objective of the guidelines was to recommend cost effective safety monitoring, highlight the preventable medication induced side effects and to facilitate the implementation of safety monitoring as a standard component of patient management. The guidelines provide an algorithm for baseline and continued treatment monitoring [3]. Whether these guidelines can be applied in different health care settings with limited resources needs to be evaluated in assessing quality of care offered to patients with BD in different settings.

Although lithium and other medicines have been in use for several decades, there is lack of information concerning treatment practices and monitoring of patients with BD in resource limited settings. In this background our study was planned with the objective of studying the adherence to safety monitoring recommendations among patients with BD attending the main tertiary care psychiatry referral clinics in the National Hospital of Sri Lanka (NHSL) in Colombo. In addition, this study also aimed to evaluate the performance of ISBD guidelines in resource limited settings and develop realistic monitoring recommendations for these settings along with strategies for implementing them. Since the ISBD criteria focus on the management of BD and emphasize on cost effective monitoring that could be applied in our local setting, recommendations given in ISBD guidelines were selected for this study.

## Methods

This study was done in all 12 specialized psychiatry follow up clinics conducted in the main tertiary care referral hospital, the NHSL, during a 2 year period from 2013 to 2015. These clinics are conducted by 4 consultants attached to the University of Colombo Psychiatry Unit and 8 consultants of the National Institute of Mental Health (NIMH), the only specialized psychiatry hospital in Sri Lanka. The target was to include a minimum of 30 consecutive patients with BD from each clinic into the study to obtain a representative sample of patients managed by all clinics in the NHSL psychiatry clinics. Ethical clearance for this study was granted by the Ethics Review Committee of the Faculty of Medicine, University of Colombo, Sri Lanka.

Monitoring of medications according to the ISBD safety monitoring recommendations was assessed, by recording the type of medication, monitoring tests and the frequency of testing. The tests considered and the recommended frequency of testing for each drug was done according to ISBD guidelines [3].

### Inclusion/exclusion criteria

Patients with a clinical diagnosis of BD diagnosed as per ICD-10 criteria who were on treatment for more than 1 year were considered eligible. Patients or guardians not giving written informed consent and those with other psychiatric diagnoses such as schizophrenia, unipolar depression, anxiety states and obsessive compulsive disorder were excluded. Demographic and clinical data, and laboratory test results were obtained from the clinic records and patient held records.

### Assessments

Patient records were retrieved and the diagnosis of each patient was confirmed before assessing the safety monitoring criteria. The recorded safety monitoring data for each test performed in every patient recruited was compared with the recommendations of the ISBD criteria pertaining to laboratory tests given in the guidelines.

### Outcomes

The percentage of patients having the tests performed at the recommended time intervals was the main outcome measure evaluated. Percentage of tests performed was compared with the studies reported from other settings. Reasons for poor monitoring were noted. Realistic safety monitoring recommendations were proposed for resource limited settings considering the existing practice, facilities and resources available.

### Statistical analysis

Percentages of patients having the tests done at the recommended time period were calculated. Associations of performing lab testing with medications used or comorbidities were determined using the chi-square test. Association of lithium measurement with mood control was also assessed. A *p* value of < 0.05 was considered as significant.

## Results

### Characteristics of the sample

Altogether 256 consecutive patients were included in the analysis. Although the target was to include 30 consecutive patients from each clinic, in some clinics this number was not achieved, as the number of patients followed up in the clinics meeting inclusion/exclusion criteria were less than 30 during the study period.

Medications used in patients with BD and characteristics and other information about the patients recorded are given in Table 1. The commonest mood stabilizer used was lithium carbonate in 164 (64.1%), followed by sodium valproate in 119 (46.5%). There were 151 (59%) patients with BD on atypical antipsychotics.

**Table 1:** Characteristics of the patients

Characteristics	Number
Number of patients, n	256
Gender, Male	137 (53.5%)
Age, years, mean (SD)	45.55 (12.41)
Age range, years	18–73
Marital Status	
Married	179 (69.9%)
Unmarried	65 (25.4%)
Separated	10 (3.9%)
Widow	2 (0.8%)
Educational level	
Below grade 5	15 (5.9%)
Between grade 5 and O/L	53 (20.7%)
Education uptoO/L examination	119 (46.5%)
Education upto A/L examination	55 (21.5%)
Graduate	10 (3.9%)
Other	4 (1.6%)
Income level	
no steady income	120 (46.9%)
has steady income	136 (53.1%)
Patients with an exacerbation of illness in the past year	
No exacerbations	136 (53.1%)
Having one or more episodes	112 (43.8%)
Missing data	8 (3.1%)
Duration on medicines, years, mean (SD)	13.17 (10.22)
Therapy	
Lithium	164 (64.1%)
Sodium valproate	118 (46.1%)
Carbamazepine	6 (2.3%)
Atypical antipsychotics	151 (59%).
Olanzepine	90 (35.2%)
Risperidone	54 (21.1%)
Quetiapine	9 (3.5%)
Typical antipsychotics	37 (14.5%)
Haloperidol	30 (11.7%)
Chlorpromazine	5 (2%)
Antidepressants	50 (19.5%)
Fluoxetine	26 (10.2%)
Imipramine	12 (4.7%)
Co-morbidities identified	
Diabetes mellitus	59 (23.0%)
Hypothyroidism	28 (10.9%)
Hypertension	41 (16.0%)
Hypercholestrolaemia	36 (14.1%)

*O/L* Ordinary Level, *A/L* Advanced level, *SD* Standard Deviation

### Monitoring of patients on lithium

The monitoring tests done and the frequency of monitoring of the patients for different medications is summarized in Table 2.

**Table 2:** Monitoring tests done and frequency of monitoring recorded for different medications

Monitoring investigation	Lithium testing percentage at 6 months, 1 year and testing frequency (*n* = 164)	Carbamazepine (*n* = 6)	Valproate (*n* = 119)	Newer antipsychotics (*n* = 151)
Serum drug concentration	75 (45.7%) in last 6 months	ND	ND	NR
	96 (58.5%) in the last year			
	Frequency – once in 13 months			
Serum Creatinine/ urea	30 (18.3%) in the last 6 months	1 record in the last year	NR	NR
	39 (23.8%) in the last year			
	Frequency – once in 33 months			
Full Blood Count (FBC)	NR	1 record in the last year	9 (7.6%) in the last year	NR
Electrolytes	34 (20.7%) in the last year	1 record in the last year	NR	NR
	Frequency – once in 5 years			
TSH	30 (18.3%) in the last year	NR	NR	NR
	Frequency – once in 5 ½ years			
Serum Calcium	ND	NR	NR	NR
Liver function tests	NR	ND	19 (16%) in the last year	NR
Fasting blood glucose	NR	NR	23 (19.3%) in the last year	31 (20.5%) in the last year
Fasting lipid profile	NR	NR	14 (11.8%) in the last year	13 (8.6%) in the last year

*NR* Not required, *ND* Not done

Of the 164 patients on lithium, only 75 patients (45.7%) had a serum lithium measurement done in the past 6 months. Lithium concentrations were recorded in the past year also only in 96 (58.5%). Average testing frequency was 0.67 tests per patient per year. Considering the tests done during 6 months and 1 year, testing frequency of lithium was once in every 13–18 months. Renal function (urea or creatinine) was available or recorded only in 39 (23.8%) during a 1 year period.

Of the patients having a serum lithium estimation done in the past year, there were 5 patients with serum lithium concentrations less than 0.4 mmol/L recorded and 2 patients having serum lithium measurements recorded above 1 mmol/L. A patient with 0.4 mmol/L serum lithium concentration had 2 tests done in the past year and one patient with a concentration above 1 mmol/L also had two tests done in the past year. The average serum lithium concentration for the patients with any serum lithium measurement was 0.67 mmol/L.

The creatinine levels were available or recorded during the last 6 months only in 30 (18.3%) patients giving a testing frequency of once in every 33 months (nearly 3 years). Thirty (18.3%) patients had Thyroid Stimulating Hormone (TSH) concentrations recorded during the past year giving a testing frequency of about once every 5 ½ years. Serum calcium concentrations were not done in any patient.

### Monitoring of patients on valproate and carbamazepine

Among the patients, 119 were on sodium valproate and6 patients were on carbamazepine. None of these patients had therapeutic blood levels recorded to establish therapeutic concentrations.

Among patients receiving sodium valproate, liver functions tests were recorded only in 19 (16%) in the past year. Full blood count was recorded only in 9 (7.6%) patients while only 23 (19.3%) had fasting blood glucose records.

Patients receiving carbamazepine therapy did not have liver enzymes tested. One patient each had full blood count, electrolyte testing and serum creatinine records.

### Monitoring of patients on atypical antipsychotics

Of the 151 patients on atypical antipsychotics only 13 (8.6%) had a lipid profile measurement done in the past year giving a testing frequency of once in every 12 years. Fasting blood glucose was also measured only in 31 (20.5%) giving a testing frequency of once in every 5 years.

### Association of monitoring with patient characteristics and medication

On statistical analysis of associations of testing with medications used and comorbidities, the patient comorbidities such diabetes or hypothyroidism influenced patient monitoring rather than therapy with medications such as lithium or atypical antipsychotics (Table 3).

**Table 3:** Associations of comorbidities/medications with monitoring tests

Factor or medication used	Monitoring	p
Diabetes	Fasting blood glucose test in the last year	<0.001
Hypothyroidism	TSH testing in the past year	<0.001
Hypercholesterolemia	Lipid profile testing in the past year	<0.001
Lithium	TSH testing in the past year	<0.05
Lithium	Serum urea or creatinine testing in the past year	<0.05
Having a disease exacerbation in the past year	Lithium monitoring in the past year	>0.05
Valproate	Liver function tested in the past year	>0.05
Atypical antipsychotics	Fasting blood glucose test in the last year	>0.05
Atypical antipsychotics	Lipid profile testing past year	>0.05

## Discussion

Monitoring of patients is an essential component of treating BD to ensure medication safety, adherence to therapy and adequate control of the disease [11]. In this study, which assessed the safety monitoring of patients with BD, the lithium concentrations were measured in the past 6 months in less than half of patients and in one-year in 59%. The minimum lithium testing frequency was once in 13 months. Of the patients having any serum lithium recorded there were few patients with serum lithium concentrations beyond the recommended therapeutic serum lithium concentrations. The average concentration of the serum lithium concentrations recorded was 0.67 mmol/L indicating that the patients who do serum lithium monitoring are also the patients achieving therapeutic serum lithium concentrations. Having poor mood control was not associated with patients having serum lithium concentration recorded, as a similar proportion of patients with good mood control and poor mood control had serum lithium estimations at 37% for the good mood control group and 38.4% for the poor mood control group. One reason for this may be the prolonged duration the patients were on treatment. For patients on lithium therapy for a long period of time, regular serum lithium concentration monitoring may not be considered necessary by the care providers, which could have contributed to lower rates of testing.

The findings of our study and similar studies done are summarized in Table 4. Our data are comparable with a veterans community study coming from the USA [7] where only 19% of patients had repeat lithium tests done at an interval of 6 months or less and a study from Brazil where only 7 of the 36 patients sampled (19%) had a lithium measurement done in the past year [8]. However our results fall short of data coming from the United Kingdom (UK) where 96% have had at least one test and 80% having 2 to 5 tests done during the preceding year. Yet, even in the UK study, 4% have had no lithium testing done at all [6].

**Table 4:** Comparison of safety monitoring tests done in the present study with previously reported studies in different settings

Therapy	Test	Sri Lanka present study (2014–15)	UK (2008–10), (Paton et al., 2013)		USA 2004–06 (Kilbourne et al., 2007)	Brazil 2009 (Souza et al., 2013)
			2008	2010		
Lithium	Serum lithium	58.5% (past year) 45.7% (past 6 months)	90% (1–4 tests in an year)	96% (1–4 tests in 1 year)	19% (past 6 months)	19% (past year)
	TFT	18.3% (Past year)	82% (1–2 tests per year)	92% (1–2 tests per year)	39% (past 6 months)	Not studied
	UC and E	23.8%: Urea and Creatinine 20.7%: Electrolytes (Past year)	81% (1–2 or more testsper year)	90% (1–2 or more tests per year)	83% (past 6 months)	Not studied
Sodium valproate	TDM	No records	Not studied	Not studied	56% (past 6 months)	Not studied
	FBC	7.6% (past year)	Not studied	Not studied	72% (past 6 months)	Not studied
	LFT	16% (past year)	Not studied	Not studied	76% (past 6 months)	Not studied
Carbamazepine	TDM	No records	Not studied	Not studied	42.9% (past 6 months)	Not studied
	FBC	1 record in the past year	Not studied	Not studied	72% (past 6 months)	Not studied
Atypical antipsychotics	FBG	20.5% (past year)	Not studied	Not studied	69% (past 6 months)	Not studied
	Lipid profile	8.6% (past year)	Not studied	Not studied	50% (past 6 months)	Not studied

*LFT* Liver function test, *FBG* Fasting blood glucose, *FBC* Full blood count, *TDM* Therapeutic Drug Monitoring, *TFT* Thyroid function test, *UC and E* Urea, Creatinine and Electrolytes

Considering the capacity of a resource limited clinic setting and possibility of some tests done being not recorded, with no electronic data monitoring, our finding of lithium monitoring of 45% in 6 months and average testing frequency every 13 months could be considered as acceptable. However, we should strive to have lithium level tested at least once in 6 months as serum lithium estimation is a low cost test which is done free of charge in Sri Lanka through the hospital laboratories for patients followed up in the clinics. These patients need to get an appointment and visit the laboratory to give blood. This test costs US Dollars (USD) 3.50–5.50 in the private sector laboratories. Considering all these factors at least one lithium estimation in 6 months in at least 80% of patients is suggested as a realistic target for our setting (Table 5).

**Table 5:** Monitoring criteria used as per ISBD guidelines and our recommendations (Ng et al., 2009)

Drug	Monitoring factor	Time interval recommended by the ISBD	Our recommendations
Lithium	Serum lithium, Electrolytes, Urea and Creatinine	Every 3–6 months	At least every 6 months in 80% patients for serum lithium and 70% for other tests
	Calcium and Thyroid Stimulating Hormone	Annually	Annually for majority of patients
Sodium valproate	Full blood count and Liver Function Tests	Annually	Annually for majority
	Therapeutic drug monitoring of sodium valproate, Fasting blood glucose and Lipid Profile	When clinically indicated	When clinically indicated
Carbamazepine	Full blood count, Liver Function Tests, Electrolytes, Urea and Creatinine	Annually	Annually for majority
	Therapeutic drug monitoring of carbamazepine	When clinically indicated	When clinically indicated
Atypical antipsychotics e.g.: Olanzapine, Risperidone, Quetiapine	Fasting blood glucose and fasting lipid profile	Annually	Annually for majority

The ISBD guidelines recommend blood urea, serum creatinine and electrolytes (renal function tests) to be measured at 3 to 6 month intervals. In our study only 30 (18.3%) patients had serum creatinine measured at 6 months and testing frequency was once in every 3 years. Electrolyte measurement was also poor with only 34 (20.7%) having it done at least once during the last year. An audit done in the USA by Kilborne *et. al.* reports of patients on lithium having serum creatinine and/or urea done at a rate of 83% at 6 month intervals [7]*.* In the UK the renal function tests were done in 70% patients at 6 month intervals [6]. Compared to the ISBD recommendations, our testing is quite low, especially considering the reported decline of renal function in patients on lithium therapy [17]. In Sri Lanka, testing serum creatinine and serum electrolytes is done free of charge through hospital laboratories and costs about USD 3 and 7 respectively in the private sector. It could be recommended to request both these tests at 6 monthly intervals every time when serum lithium is requested, to achieve a target of 70% monitoring for resource limited settings. Having boxes to be ticked for serum creatinine and electrolytes when serum lithium is tested would avoid the need for separate filling of request forms.

Our reported data on TSH measurement in only 18.3% in 1 year and frequency of once in 5 ½ years falls far short of what is done in other countries. A US study reports 39% testing during the past 6 months. Data coming from the UK show a testing rate of 66% at 6 month intervals and 92% at 1 year [6]. TSH is an expensive test in our setting if done in the private sector (approximately USD 7), but it is done free of charge in government hospitals. Measuring TSH at least annually in most patients could be recommended as a realistic target in resource limited settings.

None of the patients on lithium therapy in this study had serum calcium concentrations measured. According to ISBD recommendations, serum calcium measurement in patients with bipolar disorder is necessary as long term lithium therapy is known to cause hypercalcemia [3]. The ISBD guidelines recommend annual testing. Therefore we suggest serum calcium monitoring at least once a year in our study setting since this test is also available free of charge from hospital laboratories.

The patients who were on valproate and carbamazepine did not undergo therapeutic drug monitoring because facilities were not available through the hospitals in our study setting. The liver enzymes were also not done in any of the patients on carbamazepine while 16% of patients on valproate had liver enzymes performed. In a study done in the USA, 75% of patients on both drugs had liver function tests done [7]. In our study testing of full blood counts was also done only in 7.6% patients on valproate and on one patient on carbamazepine. In the study from USA the rate was approximately 72% for both drugs (Table 4). Although the ISBD guidelines recommend annual fasting blood glucose monitoring for patients on valproate, in our study only 19.3% had it done. Similarly renal function had been done only on a single patient on carbamazepine whereas the ISBD recommendation is to test annually. Although the monitoring of all tests were unsatisfactory we recommend annual testing for liver enzymes, full blood counts, and fasting glucose for patients on sodium valproate and carbamazepine as all these are low cost tests readily available in our setting and can be done easily with education of medical staff caring for the patients and implementation and monitoring of quality improvement programs. Therefore, more training and inputs to medical staff managing these patients is needed emphasizing the importance of these tests for monitoring.

For patients on atypical antipsychotics, the monitoring of fasting blood glucose and cholesterol levels is poor where only 20.5% had fasting blood glucose and only 8.6% had lipid profile monitoring. These levels are below the monitoring done in the USA where 69 and 50% patients had fasting blood glucose and lipid profile monitored respectively [7]. However findings of our study are comparable to some other studies done on patients taking antipsychotics. In a baseline audit done in the UK in 2006, plasma glucose and lipid profiles were measured in only 28 and 22% of patients respectively [10]. Subsequently an audit based quality improvement programme improved the monitoring of metabolic outcomes in patients taking antipsychotics. By the year 2012 the screening of blood glucose and lipid profile had doubled to 52 and 50% respectively [10]. Measuring blood glucose and lipid profiles annually and achieving monitoring in the majority of patients could be recommended as a realistic target initially in resource limited settings.

Results of our study indicate that for long-term treatment of patients on bipolar disorder, the services provided in the study setting fall below the standards demonstrated in countries such as the UK and the USA. Reasons for such lower rates of monitoring include the difficulty in implementing recommendations in the overcrowded clinics and lack of quality improvement programs and monitoring of such programs, rather than the non-availability of tests or the costs of testing. Clinical audits and monitoring of the treatment practices do not occur regularly and audit based quality improvement processes are lacking. However with establishment of healthcare quality and safety units in all major hospitals in the Ministry of Health, it is possible to implement changes with commitment of the staff. In the present psychiatric care setting, patients’ unwillingness to get the tests done would also have contributed to the lower rates. If a system is in place for regular reminders and prompting by the clinicians, a higher rate of monitoring could be achieved.

All safety monitoring tests are done free of charge in the study setting but our results indicate poor monitoring of patients. This indicates that the system in our setting is capable of providing the necessary resources to fulfill the recommended requirements of safety monitoring but patient factors and provider factors have contributed to the poor safety monitoring practices.

The safety monitoring practices and recommendations need to be modified according to the requirements, socio-cultural factors and economic conditions where the psychiatric care is provided. The guidelines recommended need to use realistic monitoring rates which could ultimately lead to better outcomes for the patients.

Quality improvement programs and clinical audits to measure performance are required to assess the situation, followed by feedback and actions taken to improve the monitoring of therapy. These should pave way for improving current practice. Several strategies were identified towards achieving this as given in Table 6.

**Table 6:** Recommendation for testing, timing intervals, physician and institutional level actions for optimal safety monitoring in resource limited settings

Drug	Physician level actions	Institutional level actions
Lithium	1.Request Serum lithium, electrolytes, and creatinine at least every 6 months 2. Educate patients on the importance of safety monitoring with blood test to overcome patient based barriers 3. Conduct clinical audits for safety monitoring 4. Request serum calcium and TSH annually 5. Record all tests done in patient held records	1.Have one form for requesting serum lithium with tick boxes for electrolytes, and creatinine 2. Ensure availability of serum lithium testing facilities at all times 3. Promote conducting clinical audits for safety monitoring and implement audit based quality improvement programmes through Healthcare quality and safety units of hospitals. 4. Make arrangements to give dates for blood testing from the clinics eliminating the need to come another day to get a test date
		5. Ensure availability of serum Calcium and TSH testing facilities at all times
Sodium valproate	1. Request full blood count, liver enzymes (AST/ALT) at baseline and annually	1. Training and continuous professional development activities for medical staff on the need for safety monitoring for alternative mood stabilizers.
	2. Request therapeutic drug monitoring (TDM) of sodium valproate, fasting glucose and lipid profiles at initiation of therapy and when clinically indicated 3. Record all tests done in patient held records	2. Establish TDM facilities for sodium valproate in government sector and inform availability to clinics
Carbamazepine	1. Request full blood count, liver enzymes (AST/ALT), electrolytes, and creatinine annually	1. Training and Continuous Professional Development (CPD) activities for medical staff on the need for safety monitoring for alternative mood stabilizers
	2. TDM of carbamazepine at initiation of therapy and when clinically indicated 3. Record all tests done in patient held records	2. Establish TDM facilities for carbamazepine in government sector and inform availability to clinics
Atypical antipsychotics e.g.: Olanzapine, Risperidone, Quetiapine	1. Request fasting blood glucose and fasting lipid profile annually 2. Record all tests done in patient held records	1. Training and CPD activities for medical staff on the need for safety monitoring for newer antipsychotics 2. Establish facilities for lipid profile testing in the government sector

In the UK, Mental Health Trusts are required to implement national guidelines and Care Quality Commission measures adherence. General Practitioners receive financial incentives when targets are met [22]. Such incentives and continuous feedback has demonstrated the improvement of monitoring of patients on lithium in the UK [6]. The audit based quality improvement programme in the UK is also an example of improvement of safety monitoring practices based on audits [10]. The quality improvement programme for patients having BD conducted a baseline audit followed up by supplementary audits. The supplementary audits revealed an increase of the proportion of patients having four serum lithium tests over the previous year from 30 to 48%, monitoring at least two tests of renal function per year from 55 to 70% and two thyroid function testing from 49 to 66% [6]. In a resource limited setting, individual commitment, feedback and incentives can lead to better outcomes to patients. A repeat study is needed in the current setting to assess impact of any interventions and the recommendations provided in this study.

Some of the clinics handled approximately 200 patients during a 6 hour period served by only 6 to 7 doctors. The low rate of recording the tests is partly due to the heavy work load. Some tests may have been performed although not recorded in clinic records. This is a limitation common to any audit, which uses recorded data. In the clinic settings no electronic data bases were maintained. There have been times during retrospective data collection, where some of the test services such as serum lithium monitoring were not available in the NHSL due to machine break down, which may have reduced the lithium testing frequency. Our sample is confined to the tertiary care referral setting in the main hospital in the capital city of the country, which has more facilities and health staff. Thus it is possible that the findings may not represent levels of monitoring in smaller units and clinics in peripheral areas of the country and may not be extrapolated to other settings.

## Conclusions

Safety monitoring of patients with BD as per the ISBD guidelines is suboptimal in our resource limited setting. Monitoring of lithium concentration is better compared to the other tests. Monitoring of valproate and carbamazepine was non-existent and the metabolic safety screening for all patients was poor. The poor rates of monitoring are mostly due to organizational factors rather than lack of resources. Realistic benchmarks for targets to be achieved and recommendations for improved monitoring are suggested considering the problems in resource limited settings, with actions aiming at healthcare professionals and for institutions.

## Data Availability

The data sets generated and analyzed during the current study are not available publicly as ethical clearance was not obtained to share data publicly. However the data is available with the corresponding author and the data are also available in the manuscript without identifying confidential patient information.

## Abbreviations

BD: Bipolar Disorder
CPD: Continuous Professional Development
ISBD: International Society for Bipolar Disorders
NHSL: National Hospital of Sri Lanka
NIMH: National Institute of Mental Health
TDM: Therapeutic Drug Monitoring
TSH: Thyroid Stimulating Hormone
UK: United Kingdom
USA: United States of America
USD: United States Dollars
